# Supplementary material for: Discontinuous transition to active nematic turbulence
Source: Nat Commun. 2025 Dec 15;16:11169. doi: 10.1038/s41467-025-67499-6 (PMC12708656; doi:10.1038/s41467-025-67499-6)
Supplement: Supplementary file 1 — Supplementary Information [file 41467_2025_67499_MOESM1_ESM.pdf]

Supporting Information
Discontinuous transition to active nematic turbulence

Malcolm Hillebrand[1,2,3] and Ricard Alert[1,2,4,5,6,7,*]

[1]*Max Planck Institute for the Physics of Complex Systems, Nöthnitzerst. 38, 01187 Dresden, Germany*
[2]*Center for Systems Biology Dresden, Pfotenhauerst. 108, 01307 Dresden, Germany*
[3]*Department of Mathematics and Applied Mathematics, University of Cape Town, Rondebosch 7701, South Africa*
[4]*Cluster of Excellence Physics of Life, TU Dresden, 01062 Dresden, Germany*
[5]*Departament de Física de la Matèria Condensada, Universitat de Barcelona, Martí i Franquès 1, 08028 Barcelona, Spain*
[6]*Universitat de Barcelona Institute of Complex Systems (UBICS), Barcelona, Spain*
[7]*Institució Catalana de Recerca i Estudis Avançats (ICREA), Barcelona, Spain*

(Dated: November 19, 2025)

SUPPLEMENTARY FIGURES

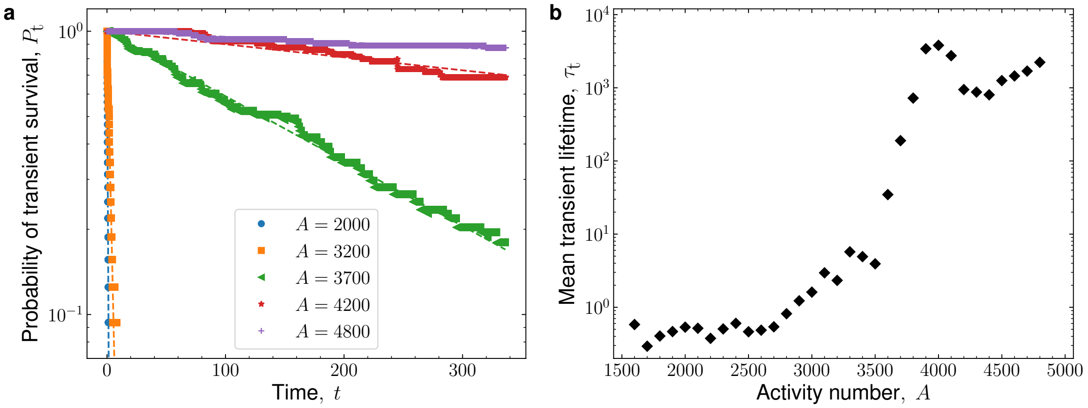

Figure S1 | Lifetimes of chaotic transients. a, The probability P_t of a chaotic transient surviving a certain time t decays exponentially for different values of the activity number A. The dashed lines indicate a fit to an exponential function $P_\mathrm{t} = \exp(-t/\tau_\mathrm{t})$, from which we obtain the lifetime τ_t. **b**, The mean lifetime τ_t of chaotic transients increases very sharply (by orders of magnitude) as the activity number A approaches the region of the transition to turbulence, which begins around $A \approx 3800$. The unit of time in this figure is τ_r, as defined in the Main Text.

* ricard.alert@ub.edu

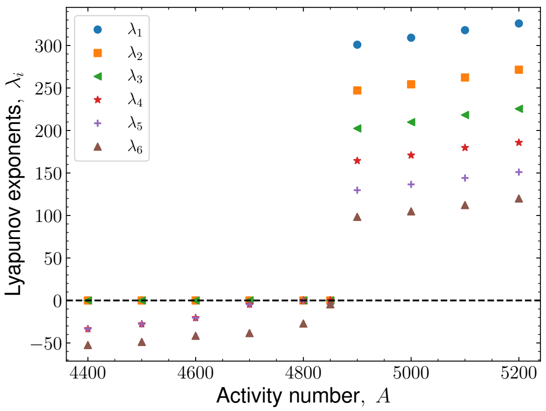

Figure S2 | The spectrum of Lyapunov exponents reveals a sharp transition to high-dimensional chaos. The largest six Lyapunov exponents measured after a time $t_\mathrm{f} = 5000\tau_\mathrm{r}$ for a single realization. Below the critical activity $A^* \approx 4900$, all Lyapunov exponents are zero or negative. As the stable laminar state approaches the transition to chaos, negative Lyapunov exponents approach zero. Immediately following the transition, at least six Lyapunov exponents are positive, indicating a high-dimensional chaotic attractor. Thus, high-dimensional chaos seems to set in directly, without going through a region of low-dimensional chaos.

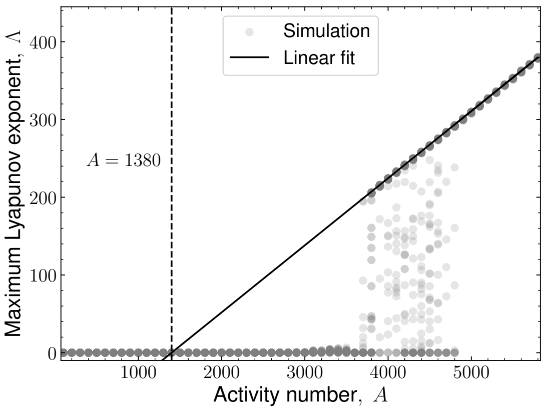

Figure S3 | Linear fit of the increase of the maximum Lyapunov exponent (MLE) with activity. The straight line fitted to the positive MLE values allows us to extrapolate the trend back to an intersection with $\Lambda = 0$ at $A = 1380$, which suggests that chaotic transients can already start appearing at such low activities.

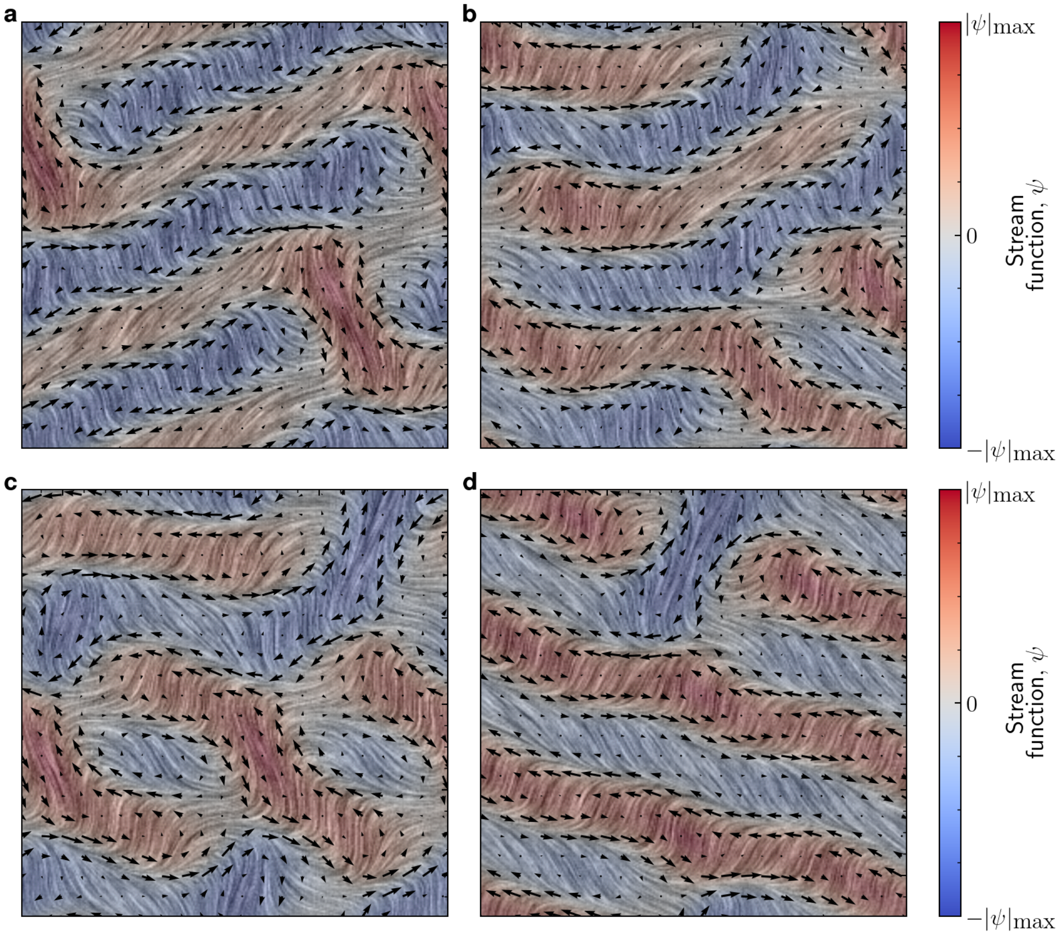

Figure S4 | Multiple vortex solutions are possible at the same activity. a-d, Four representative snapshots of the stream function (color) and velocity field (arrows) overlaid on the nematic director (gray line integral convolution texture) at activity number $A = 2400$. Each case results from a different seeding of random noise for the initial transient (see Methods).

SUPPLEMENTARY MOVIES

Supplementary Movie 1: Oscillating vortex state. A representative oscillating solution at $A = 1560$, with the flow switching between vortex states with a regular period. The movie displays both the nematic director field (grey underlying texture), and the flow field (velocity shown by arrows, stream function by color).

Supplementary Movie 2: Active turbulence. Simulation of the active nematic in the turbulent regime, $A = 6000$, showing the chaotic rearrangements of the nematic director field (grey underlying texture) and the flow field (velocity shown by arrows, stream function by color).

Supplementary Movie 3: Chaotic transient. A three-vortex state becomes unstable and exhibits growing oscillations followed by transient chaos where the flow rearranges rapidly, exploring many configurations, before finding a final steady state through oscillating relaxation. The movie displays both the nematic director field (grey underlying texture), and the flow field (velocity shown by arrows, stream function by color).
