# Supplementary material for: Discontinuous transition to active nematic turbulence
Source: Nat Commun. 2025 Dec 15;16:11169. doi: 10.1038/s41467-025-67499-6 (PMC12708656; doi:10.1038/s41467-025-67499-6)
Supplement: Supplementary file 2 — Description of Additional Supplementary Files [file 41467_2025_67499_MOESM2_ESM.pdf]

Description of Additional Supplementary Files

Supplementary Movie 1: Oscillating vortex state. A representative oscillating solution at A = 1560, with the flow switching between vortex states with a regular period. The movie displays both the nematic director field (grey underlying texture), and the flow field (velocity shown by arrows, stream function by color).

Supplementary Movie 2: Active turbulence. Simulation of the active nematic in the turbulent regime, A = 6000, showing the chaotic rearrangements of the nematic director field (grey underlying texture) and the flow field (velocity shown by arrows, stream function by color).

Supplementary Movie 3: Chaotic transient. A three-vortex state becomes unstable and exhibits growing oscillations followed by transient chaos where the flow rearranges rapidly, exploring many configurations, before finding a final steady state through oscillating relaxation. The movie displays both the nematic director field (grey underlying texture), and the flow field (velocity shown by arrows, stream function by color).
